# PGC-1*α* Protects against Hepatic Ischemia Reperfusion Injury by Activating PPAR*α* and PPAR*γ* and Regulating ROS Production

**DOI:** 10.1155/2021/6677955

**Published:** 2021-05-19

**Authors:** Chaoqun Wang, Zihao Li, Baolei Zhao, Yaohua Wu, Yao Fu, Kai Kang, Yao Li, Liqian Dong, Xiaozhuang Li, Bao Zhang, Huibo Wu, Benqiang Shen, Yanan Xu, Shangha Pan, Hongchi Jiang, Dawei Wang, Yong Ma

**Affiliations:** ^1^Key Laboratory of Hepatosplenic Surgery, Ministry of Education, Department of General Surgery, The First Affiliated Hospital of Harbin Medical University, Harbin 150001, China; ^2^Department of Hepatobiliary Surgery, The Affiliated Hospital of Binzhou Medical University, Binzhou 256600, China; ^3^Department of Ultrasound, The First Affiliated Hospital of Harbin Medical University, Harbin 150001, China; ^4^Department of Intensive Care Unit, The First Affiliated Hospital of Harbin Medical University, Harbin 150001, China

## Abstract

Peroxisome proliferator-activated receptors (PPARs) *α* and *γ* have been shown to be protective in hepatic ischemia/reperfusion (I/R) injury. However, the precise role of PPAR*γ* coactivator-1*α* (PGC-1*α*), which can coactivate both of these receptors, in hepatic I/R injury, remains largely unknown. This study was designed to test our hypothesis that PGC-1*α* is protective during hepatic I/R injury in vitro and in vivo. Our results show that endogenous PGC-1*α* is basally expressed in normal livers and is moderately increased by I/R. Ectopic PGC-1*α* protects against hepatic I/R and hepatocyte anoxia/reoxygenation (A/R) injuries, whereas knockdown of endogenous PGC-1*α* aggravates such injuries, as evidenced by assessment of the levels of serum aminotransferases and inflammatory cytokines, necrosis, apoptosis, cell viability, and histological examination. The EMSA assay shows that the activation of PPAR*α* and PPAR*γ* is increased or decreased by the overexpression or knockdown of PGC-1*α*, respectively, during hepatic I/R and hepatocyte A/R injuries. In addition, the administration of specific antagonists of either PPAR*α* (MK886) or PPAR*γ* (GW9662) can effectively decrease the protective effect of PGC-1*α* against hepatic I/R and hepatocyte A/R injuries. We also demonstrate an important regulatory role of PGC-1*α* in reactive oxygen species (ROS) metabolism during hepatic I/R, which is correlated with the induction of ROS-detoxifying enzymes and is also dependent on the activations of PPAR*α* and PPAR*γ*. These data demonstrate that PGC-1*α* protects against hepatic I/R injury, mainly by regulating the activation of PPAR*α* and PPAR*γ*. Thus, PGC-1*α* may be a promising therapeutic target for the protection of the liver against I/R injury.

## 1. Introduction

Hepatic ischemia/reperfusion (I/R) injury is a common complication of transplantation, liver resection, and various types of shock, especially hemorrhagic shock. In addition to cell apoptosis and necrosis, severe I/R injury can cause a dysregulated systemic inflammatory response that culminates in multiple organ dysfunction. The current settlement for hepatic I/R injury is merely supportive care, and thus, new therapeutic strategies are urgently needed [1, 2]. Moreover, an understanding of the molecular mechanisms underlying hepatic I/R injury will provide the basis for the development of new strategies for preventing liver injury and improving the prognosis in these patients.

Recently, peroxisome proliferator-activated receptor *γ* (PPAR*γ*), a member of the nuclear hormonal receptor superfamily, has been shown to be an important endogenous regulator and a potential therapeutic target for ischemic liver injury [3–5]. The PPAR*γ* coactivator-1*α* (PGC-1*α*), as its name implies, is known to be a transcriptional coactivator for PPAR*γ*. PGC-1*α* was first identified through its functional interaction with PPAR*γ* in brown adipose tissue (BAT), a mitochondria-rich tissue specialized for thermogenesis [6]. Following its discovery as a PPAR*γ* coactivator, several PGC-1*α* target nuclear receptors have been identified, including PPAR*α*, which is a key moderator in mitochondrial fatty acid oxidation and an important regulator of hepatic I/R injury [7–9]. It has been demonstrated that PPAR*α* is a key regulator of the hepatic inflammatory response to I/R, and its activation in cultured hepatocytes is directly protective against oxidative injury [8]. Although PPAR*α* and PPAR*γ* have been shown to be protective in liver I/R injury, the precise role of PGC-1*α*, which can coactivate both of these targets, has not been investigated to date.

In addition, excessive reactive oxygen species (ROS) generation has proved to be critical in the pathogenesis of I/R-induced liver injury, which leads to oxidative stress, causing inflammation and cellular damage and death [10, 11]. Recently, increasing evidence has indicated that PGC-1*α* is a promising regulator of ROS metabolism [12–15]. Sánchez-Ramos et al. have reported that knockout mice were more sensitive to hepatic I/R damage, especially for the steatotic one, which is likely associated with its capacity to induce antioxidant gene expression. However, the effect of ectopic PGC-1*α* and the relationship among PGC-1*α*, PPARs, and ROS metabolism in this process still remain unclear.

The above findings prompted us to hypothesize that the ectopic PGC-1*α* protein is protective and PGC-1*α* can be a promising therapeutic target for the treatment of the liver against I/R injury. Therefore, the current study was designed to test our hypothesis by using a mouse partial hepatic I/R injury model. The in vitro effects of PGC-1*α* on hepatocyte anoxia/reoxygenation (A/R) injury were also tested.

## 2. Materials and Methods

### 2.1. Animals

Male 8-week-old C57BL/6 (18–20 g) mice were supplied by the Animal Research Center at the First Clinical Medical School of Harbin Medical University (Harbin, China). All surgical procedures and care administered to the animals were approved by the Institutional Animal Care and Use committee of Harbin Medical University.

### 2.2. Construction of Adenoviral (Ad) Vectors

The adenoviral vector for the expression of PGC-1*α* (Ad-PGC-1*α*) or GFP alone (Ad-GFP) was a kind gift from Daniel P. Kelly (Washington University School of Medicine, St. Louis, MO). We generated the PGC-1*α* shRNA adenoviral vector (Ad-shPGC-1*α*) using an effective sequence and its negative control Ad-shScramble, as described previously [16–18].

### 2.3. Mouse Hepatic I/R Model

A previously characterized 70% hepatic warm I/R injury model was performed on mice [19]. In certain groups, the animals were infused 1.5 h prior to the onset of liver ischemia with a single dose of MK886 (a specific PPAR*α* antagonist, 3 mg/kg, IP) or GW9662 (a specific PPAR*γ* antagonist, 1 mg/kg, IP). The mice were sacrificed at 3 h, 6 h, 12 h, and 24 h after reperfusion, and the liver and serum samples were collected for further analyses, including serum analyses, histological examination [20], quantitative RT-PCR analysis, electrophoretic mobility shift assay (EMSA) [21], western blotting, terminal deoxynucleotidyl transferase-mediated dUTP nick-end labeling (TUNEL) assay, DNA fragmentation ELISA, caspase-3 activity assay, ROS detection [22], assessment of malondialdehyde (MDA), and 4-hydroxynonenal (HNE) contents [23] and measurements of superoxide dismutase (SOD), catalase (CAT), and glutathione peroxidase (GPX) activities [24].

### 2.4. Hepatocytes Isolation and A/R Assay

Mouse hepatocytes were isolated by a modified in situ collagenase perfusion technique as described previously [19]. To simulate liver I/R, hepatocytes were incubated under hypoxic conditions as described previously [19, 20]. In certain groups, the hepatocytes were also pretreated with MK886 (10 *μ*M), GW9662 (5 *μ*M), or N-acetyl-L-cysteine (NAC) (10 mM) 1 h before the onset of A/R. At 24 h after A/R, the live cells were first examined by fluorescence microscopy, after which cell viability, necrosis, apoptosis, and ROS detection assays were performed as described previously [22, 25].

### 2.5. Quantitative RT-PCR Analysis

RNA was extracted according to the manufacturer's instructions with the RNeasy kit (Qiagen, Valencia, CA, USA). cDNA was prepared with the TaqMan Reverse Transcription Reagents Kit using random hexamers (PE Applied Biosystems, Foster City, CA). Quantitative PCR was performed on a sequence detection system (Prism model 7500; ABI) using the *ΔΔ*CT method, which provides normalized expression values. The relative mRNA expression levels were calculated by comparing the target genes to glyceraldehyde-3-phosphate dehydrogenase (GAPDH) mRNA, respectively, and were expressed as means ± SD of the fold change relative to the control, which was set at 1. The probe and primer sets utilized in this study are summarized in Supplementary Table 1.

### 2.6. Statistical Evaluation

All data are expressed as the mean ± SD. Significant differences between the groups were determined by ANOVA, with a Bonferroni correction for continuous variable and multiple groups. Two-tailed Student's *t* test was used for the comparison of a normally distributed continuous variable between 2 groups. The level of significance was set at a *P* value less than 0.05 for all analyses.

For further details of experimental materials and procedures, please see the Supplementary files (available here).

## 3. Results

### 3.1. The Hepatic Expression of PGC-1*α* Is Increased by I/R, and Virus Vectors Are Effective for Achieving Its Expression Modification in the Liver

**Figure 1:**
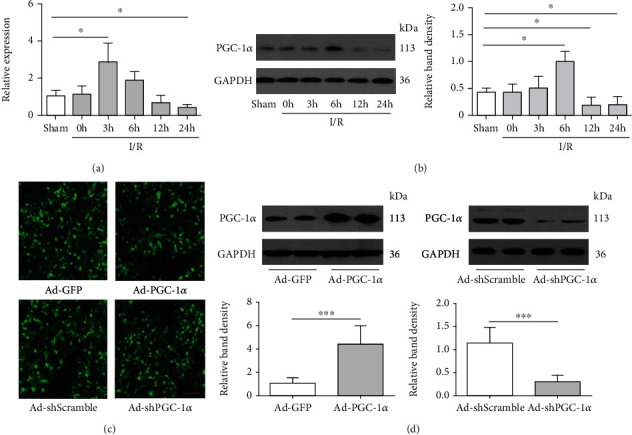
Hepatic I/R enhances the in situ expression of PGC-1*α*, and transduction with adenoviral (Ad) vectors is effective for inducing intrahepatic PGC-1*α* overexpression or knockdown. (a) The mRNA expression of PGC-1*α* in the mouse liver of each group was assessed by real-time RT-PCR (*n* = 3). (b) The protein expression of PGC-1*α* was detected by western blot. (c) Fluorescent microscopy showed the efficient transduction of hepatocytes, as indicated by GFP expression, 72 h following administration of the Ad vectors. (d) PGC-1*α* protein expression in the mouse liver of each group was assessed by western blot analysis. Bar: 100 *μ*m. ^∗^*P* < 0.05, ^∗∗^*P* < 0.01, and ^∗∗∗^*P* < 0.001. The results are representative of three independent experiments.

**Figure 2:**
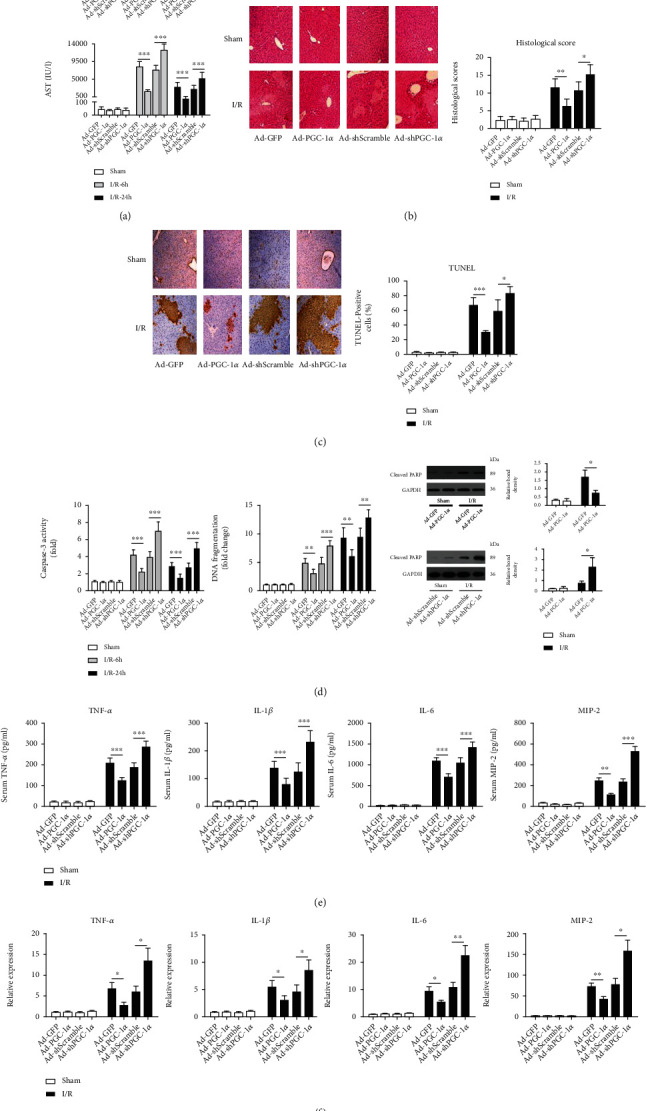
PGC-1*α* protects the liver against I/R injury. (a) Serum levels of aminotransferases (ALT and AST) were detected in the mice subjected to Ad-GFP, Ad-PGC-1*α*, Ad-shScramble, and Ad-shPGC-1*α* at 6 h and 24 h after liver I/R (*n* = 6). (b) Representative images (200x magnification) of H&E-stained liver sections (6 h after I/R) were taken, and histopathological scoring of hepatic injury was performed. (c) Representative images (200x magnification) of liver sections (6 h after I/R) stained by TUNEL were taken, and TUNEL-positive cells were counted as described in Materials and Methods. (d) Caspase-3 activity, DNA fragmentation, and cleaved PARP expression in the mouse livers were assessed by ELISA and western blot (*n* = 3-6). (e) Systemic TNF-*α*, IL-1*β*, IL-6, and MIP-2 levels at 6 h after liver I/R were measured by ELISA (*n* = 6). (f) The relative mRNA expression levels of TNF-*α*, IL-1*β*, IL-6, and MIP-2 in the mouse liver tissues at 6 h after I/R were determined by quantitative RT-PCR (*n* = 3). ^∗^*P* < 0.05, ^∗∗^*P* < 0.01, and ^∗∗∗^*P* < 0.001.

To determine whether PGC-1*α* plays a role in hepatic I/R injury, we first examined its expression in hepatic lobes that had been subjected to 75 min of ischemia and 0 h, 3 h, 6 h, 12 h, and 24 h of reperfusion in mice, respectively. As expected, hepatic I/R briefly stimulated the hepatic expression of PGC-1*α* from 3 hours after the initiation of reperfusion up to 6 hours and then declined thereafter (Figures 1(a) and 1(b)). To further determine the role of PGC-1*α* in liver I/R injury, we used gain- and loss-of-function strategies to overexpress or downregulate the intrahepatic PGC-1*α* by tail vein injection with Ad-PGC-1*α* or Ad-shPGC-1*α* (1 × 10^9^ TCID50 per mouse), respectively. Three days following gene transfer, the mice were subjected to partial hepatic I/R, as described in Materials and Methods. To verify the efficiency of the transduction, parts of the collected liver tissues in the sham group were fixed in 4% paraformaldehyde followed by an overnight incubation in 30% sucrose and then embedding in OCT compound for GFP visualization under fluorescence microscope (Figure 1(c)). Western blotting was used to document PGC-1*α* expression simultaneously, in order to further demonstrate the transduction of the Ad vectors. The PGC-1*α* expression in Ad-PGC-1*α*-treated livers was significantly higher than in Ad-GFP-treated livers. Ad-shPGC-1*α* successfully decreased the expression of PGC-1*α*, compared with Ad-shScramble-treated livers. There was no difference between Ad-GFP- and Ad-shScramble-treated livers (Figure 1(d)). The serological and histological examinations showed the maintenance of normal levels of serum markers of liver function (Figure 2(a)) and the absence of any acute inflammation, fibrosis, apoptosis, necrosis, or overt histological evidence of toxicity (Figures 2(b) and 2(c)). These data indicate that adenovirus vectors are an effective nontoxic means to achieve intrahepatic PGC-1*α* overexpression and knockdown.

### 3.2. PGC-1*α* Protects against Hepatic I/R Injury in Mice

The mice were subjected to 75 min of 70% hepatic ischemia or to a sham operation. To evaluate the severity of the hepatic I/R injury, we first measured the levels of ALT and AST in sera, which are sensitive indicators of liver damage. Hepatic I/R caused significant increases in the levels of ALT and AST 6 h and 24 h after the operation, in comparison with the sham-operated mice. The ectopic expression of PGC-1*α* significantly reduced the degree of ALT and AST increases, whereas knockdown of endogenous PGC-1*α* revealed the opposite effects, in comparison with their corresponding control mice (Figure 2(a)). HE staining exhibited that the sham operation did not show any effects on liver histology (Figure 2(b)). Histological alteration in the livers from Ad-GFP- or Ad-shScramble-transduced I/R mice was characterized as hemorrhagic change, inflammatory cell infiltration and focal necrosis. In contrast, the overexpression of PGC-1*α* ameliorated such pathological changes significantly, whereas the PGC-1*α* knockdown had an aggravating effect on these changes (Figure 2(b)). There was no significant difference between the Ad-GFP- and Ad-shScramble-treated livers.

The injury scores of the mouse livers in the Ad-GFP- or Ad-shScramble-transduced I/R group were significantly higher than those in the sham group. Additionally, PGC-1*α* overexpression significantly decreased the histological scores, whereas the knockdown of endogenous PGC-1*α* had the reverse effect, in comparison with the corresponding control I/R mice (Figure 2(b)). We also detected the levels of apoptosis and necrosis by TUNEL assay, caspase-3 activity assay, and DNA fragmentation ELISA. The results of the TUNEL-positive cell counting and representative images are shown in Figure 2(c). While almost no TUNEL-positive cells were detected in the sham control, hepatic I/R induced a percentage of TUNEL-positive cells of 66.67% ± 11.11%, which was decreased to 25.67% ± 6.56% by PGC-1*α* overexpression and increased to 82.50% ± 9.85% by PGC-1*α* knockdown (Figure 2(c)). There was no significant difference between the Ad-GFP- and Ad-shScramble-treated livers. An increase in caspase-3 activity and DNA fragmentation were observed in the I/R groups. Ectopic PGC-1*α* expression significantly inhibited the increase, whereas the knockdown of endogenous PGC-1*α* showed the opposite effects (Figure 2(d)). To further confirm that I/R induced apoptosis, we measured the accumulation of cleaved PARP, an important apoptosis marker [26], using western blot analysis. As expected, the mice subjected to liver I/R had significantly increased cleaved PARP levels compared to the sham groups. PGC-1*α* overexpression and knockdown significantly decreased and increased the cleaved PARP levels, respectively, in comparison with the corresponding control I/R mice (Figure 2(d)).

### 3.3. PGC-1a Alleviates the Inflammatory Response to Hepatic I/R in Mice

The serum levels of inflammatory cytokines and chemokines that have been shown to play crucial roles in the pathophysiology of hepatic I/R injury, including TNF-*α*, IL-1*β*, IL-6, and MIP-2, were detected at the same time [27–29]. As shown in Figure 2(e), hepatic I/R significantly increased the levels of cytokines and chemokines, compared with the sham group. Overexpression of PGC-1*α* attenuated these increases as the levels of the cytokines and chemokines were significantly lower than those in the untreated hepatic I/R mice. PGC-1*α* knockdown had the opposite effect, as evidenced by further increases in the levels of the 4 parameters (Figure 2(e)). Furthermore, we also assessed the mRNA expression profiles of these inflammatory cytokines and chemokines in the ischemic livers. The mRNA expression levels of TNF-*α*, IL-1*β*, IL-6, and MIP-2 were significantly higher in the hepatic I/R mice than in the sham control mice (Figure 2(f)). PGC-1*α* overexpression decreased the hepatic I/R-induced high levels of these 4 parameters significantly, whereas PGC-1*α* knockdown caused a further elevation.

### 3.4. PGC-1*α* Increases the Activation of PPAR*α* and PPAR*γ* In Vivo

**Figure 3:**
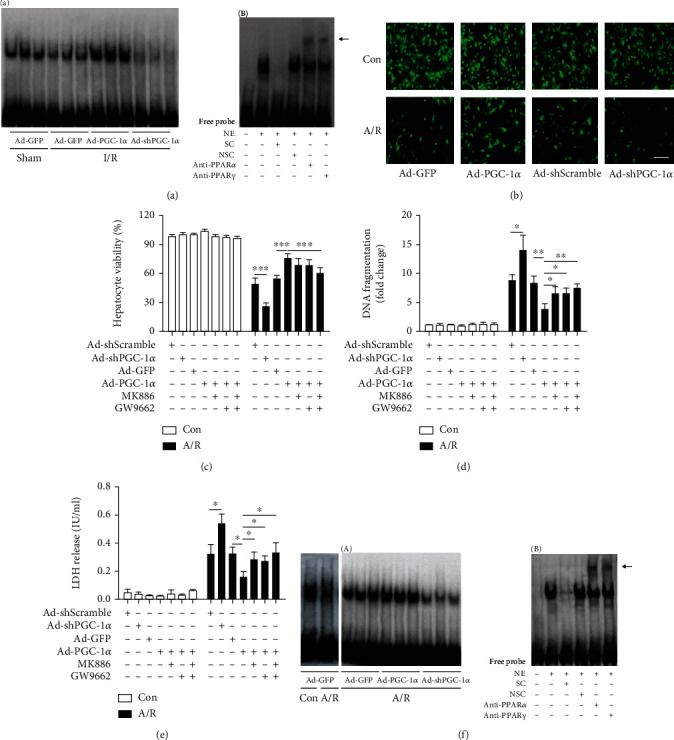
PGC-1*α* regulates the activation of PPAR*α* and PPAR*γ* in vivo and in vitro and protects A/R-induced hepatocyte injury. (a) The activation of PPAR*α* and PPAR*γ* in liver tissues was assessed in the mice subjected to Ad-PGC-1*α*, Ad-shPGC-1*α*, and the related control at 6 h after liver I/R by EMSA (A), and the specificity of the PPAR*α* and PPAR*γ* bands were confirmed with competition and supershift assays; the supershift bands are indicated by the arrow (B). (b) Representative fluorescence microscopy images of the hepatocytes from certain groups were taken at 24 h after A/R injury. (c) Cell viability was determined at 24 h after reoxygenation by a CCK-8 assay. (d) DNA fragmentation was determined at 24 h after reoxygenation. (e) LDH release was measured in each group. (f) The activation of PPAR*α* and PPAR*γ* in the hepatocytes was assessed by EMSA (A), and the specificity of the PPAR *α* and *γ* bands was also confirmed (B). The data was presented as the means ± SD of three independent experiments. Bar: 200 *μ*m, ^∗^*P* < 0.05, ^∗∗^*P* < 0.01, and ^∗∗∗^*P* < 0.001. NE: nuclear extracts; SC: specific competition; NSC: nonspecific competition; anti-PPAR*α*: PPAR*α* antibody; anti-PPAR*γ*: PPAR*γ* antibody.

To determine the effects of overexpression or knockdown of intrahepatic PGC-1*α* on the activation of PPAR*α* and PPAR*γ*, liver nuclear extracts were subjected to EMSA. As shown in Figure 3(a), after 75 min of ischemia and 6 h of reperfusion, the activation of the PPARs in the Ad-GFP-transduced I/R group was mildly decreased, and PGC-1*α* overexpression or knockdown attenuated or aggravated this reduction, respectively, in comparison with Ad-GFP-transduced sham mice (Figure 3(a)). The specificities of the PPAR*α* and PPAR*γ* bands were confirmed with competition and supershift assays. As shown in Figure 3(a), the shifted bands were clearly visible. The unlabeled specific probe competed efficiently with the labeled probe, while the nonspecific competitor was unable to compete with the labeled probe, indicating the specificity of the protein-DNA interaction. There was no difference between the Ad-GFP- and Ad-shScramble-treated livers (data not shown). Together, our results indicate that PGC-1*α* increases the activation of PPAR*α* and PPAR*γ* in liver I/R injury.

### 3.5. PGC-1*α* Protects A/R-Induced Hepatocyte Injury

To avoid the influence of the complicated environment in vivo and to further confirm the hepatoprotective effect of PGC-1*α*, we performed an in vitro experiment using a primary hepatocyte A/R assay, as described previously [19, 30]. As shown in Figure 3(b), after 4 h of anoxia and 24 h of reoxygenation, the number of visible live hepatocytes in the Ad-GFP- or Ad-shScramble-transduced A/R group was dramatically decreased. Ectopic expression of PGC-1*α* increased the number significantly, whereas knockdown of endogenous PGC-1*α* had the opposite effect, in comparison with their corresponding control hepatocytes. In accordance with these data, a CCK-8 assay showed that A/R reduced the hepatocyte viability by approximately 45% in the Ad-GFP group (Figure 3(c)). Similar results were also observed in the Ad-Scramble group. However, PGC-1*α* knockdown further decreased cell viability to 25.83 ± 3.66%. In contrast, PGC-1*α* overexpression significantly rescued the decreased cell viability caused by A/R, compared with Ad-GFP-transduced hepatocytes (75.33 ± 5.32% versus 53.83 ± 4.45%; Figure 3(c)) and thus appeared to be protective. A subsequent analysis of A/R-induced cell death revealed that the effects of PGC-1*α* were similar to the in vivo findings. As shown in Figure 3(d), A/R induced an 8.23 ± 1.35-fold increase in DNA fragmentation in the Ad-GFP cells, which was decreased to 3.71 ± 1.02 by PGC-1*α* overexpression (Figure 3(d)). Conversely, PGC-1*α* knockdown increased the DNA fragmentation significantly, from 8.67 ± 0.99 to 13.87 ± 2.63%, in comparison with the Ad-shScramble cells (Figure 3(d)). There was no difference between the Ad-GFP- and Ad-shScramble-treated livers. Similar results were also observed by measuring lactate dehydrogenase (LDH) (Figure 3(e)). Taken together, the aforementioned results indicate that PGC-1*α* has a protective role in A/R-induced hepatocyte injury.

### 3.6. PGC-1*α* Increases the Activation of PPAR*α* and PPAR*γ* In Vitro

We next assessed if PGC-1*α* increases the activation of PPAR*α* and PPAR*γ* in vitro. Nuclear extracts of primary hepatocytes were obtained and analyzed by EMSA. The changes in the activation of the PPARs were similar to the in vivo findings. As shown in Figure 3(f), after 4 h of anoxia and 24 h of reoxygenation, activation of the PPARs in the Ad-GFP-transduced hepatocytes was significantly decreased, compared with Ad-GFP-transduced control. PGC-1*α* overexpression or knockdown attenuated or aggravated this reduction, respectively, in comparison with the Ad-GFP-transduced A/R hepatocytes. The specificities of the PPAR bands were also confirmed with competition and supershift assays (Figure 3(f)). There was no difference between the Ad-GFP- and Ad-shScramble-treated livers (data not shown). Together, our results indicate that PGC-1*α* increases the activation of PPAR*α* and PPAR*γ* in hepatocyte A/R injury.

### 3.7. Inhibition of Either PPAR*α* Or PPAR*γ* Alleviates the Hepatoprotective Effect of PGC-1*α* In Vitro and In Vivo

**Figure 4:**
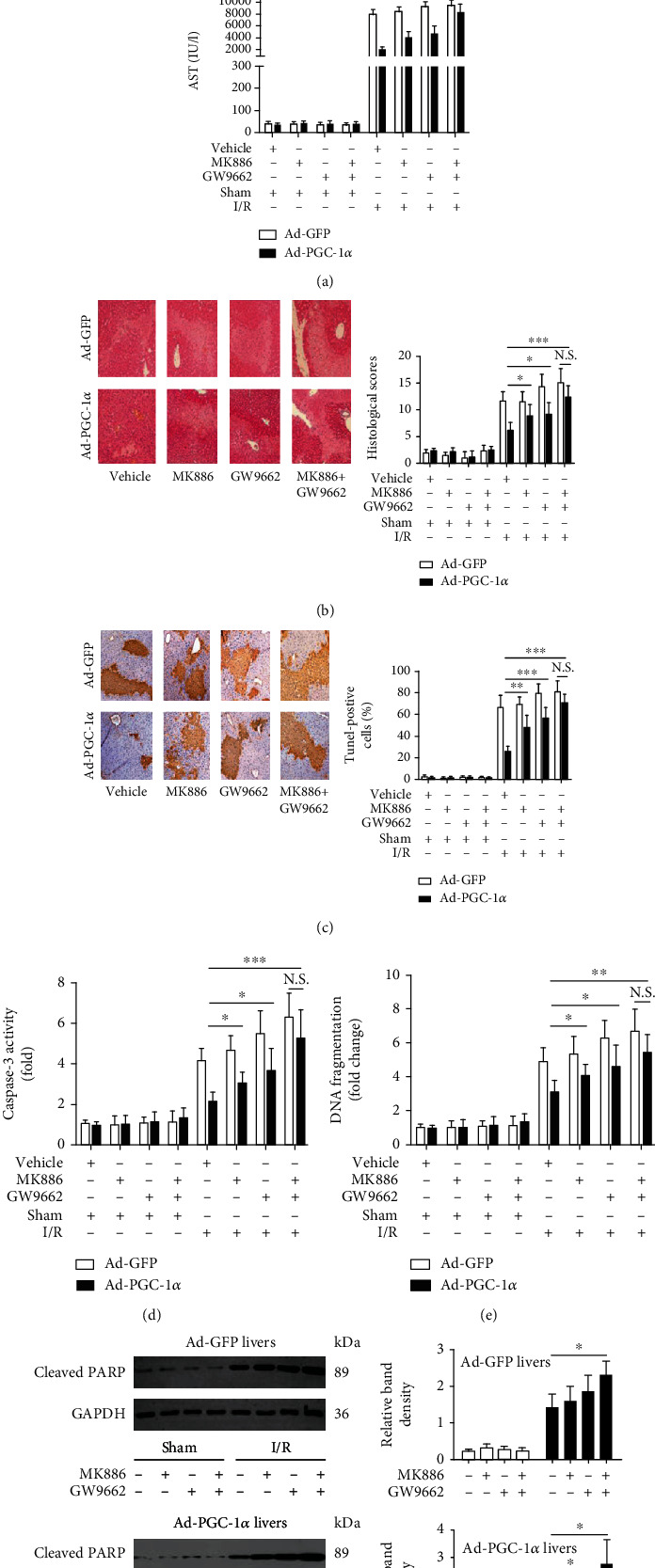
Inhibition of either PPAR*α* or PPAR*γ* alleviates the hepatoprotective effect of PGC-1*α* in vivo. (a) The serum levels of aminotransferases were measured in the mice subjected to Ad-PGC-1*α*, Ad-PGC-1*α*+MK886, Ad-PGC-1*α*+GW9662, and Ad-PGC-1*α*+MK886+GW9662 at 6 h after liver I/R (*n* = 6). (b) Representative images (200x magnification) of the H&E-stained liver sections (6 h after I/R) were taken, and histopathological scoring of hepatic injury was performed (*n* = 6). (c) Representative images (200x magnification) of the liver sections (6 h after I/R) stained by TUNEL were taken, and TUNEL-positive cells were counted as described in Materials and Methods (*n* = 6). (d–f) Caspase-3 activity, DNA fragmentation, and cleaved PARP expression in the mouse livers of each group were assessed by ELISA and western blot analysis at 6 h after I/R (*n* = 3-6). ^∗^*P* < 0.05, ^∗∗^*P* < 0.01, and ^∗∗∗^*P* < 0.001.

**Figure 5:**
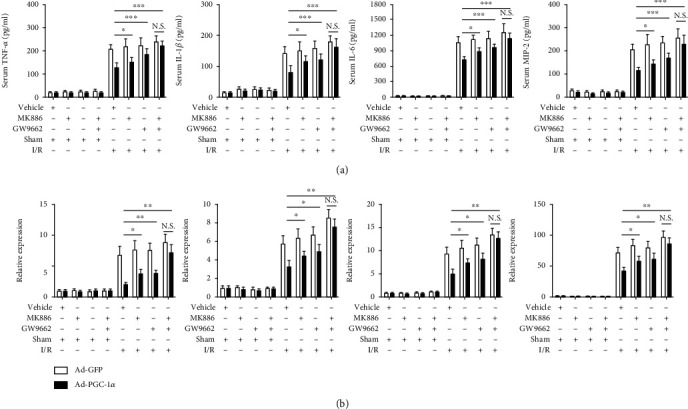
Inhibition of either PPAR*α* or PPAR*γ* alleviates the effect of PGC-1*α* on inflammatory mediator production induced by liver I/R injury. (a) Systemic TNF-*α*, IL-1*β*, IL-6, and MIP-2 levels were assessed at 6 h after liver I/R by ELISA. (b) the relative mRNA expression levels of TNF-*α*, IL-1*β*, IL-6, and MIP-2 in the mouse liver tissues were determined by qRT-PCR at 6 h after liver I/R. The data are expressed as the mean ± SD of 6 animals per group. ^∗^*P* < 0.05, ^∗∗^*P* < 0.01, and ^∗∗∗^*P* < 0.001.

To further examine whether the hepatoprotective effects of PGC-1*α* during I/R was due to the increased activation of PPAR*α* and PPAR*γ*, both the Ad-PGC-1*α*-transduced primary hepatocytes and the mice were treated with specific antagonists of PPAR*α* (MK886) or PPAR*γ* (GW9662), respectively. As shown in Figures 3(c)–3(e), both MK886 and GW9662 pretreatment could reduce the hepatoprotective effects of PGC-1*α* overexpression in vitro, compared with the Ad-PGC-1*α*-transduced alone groups, as indicated by the decreased levels of hepatocyte viability and increased levels of cell death. Next, we used the murine models to evaluate such effects in vivo (Figure 4). As expected, both MK886 and GW9662 pretreatment could reduce the hepatoprotective effects of PGC-1*α* overexpression in vivo, compared with the Ad-PGC-1*α*-transduced alone groups, as indicated by the increased levels of serum aminotransferases (Figure 4(a)), histological changes (Figures 4(b) and 4(c)), necrosis, and apoptosis (Figure 4(d)), as well as by the production of inflammatory cytokines and chemokines (Figure 5). Together, these data indicate that PPAR*α* and PPAR*γ* play important roles in the hepatoprotective effect of PGC-1*α* in vitro and in vivo.

### 3.8. PGC-1*α* Regulates ROS Metabolism during Hepatic I/R and Hepatocyte A/R Injuries and Decreases Liver I/R-Induced Oxidative Damage

**Figure 6:**
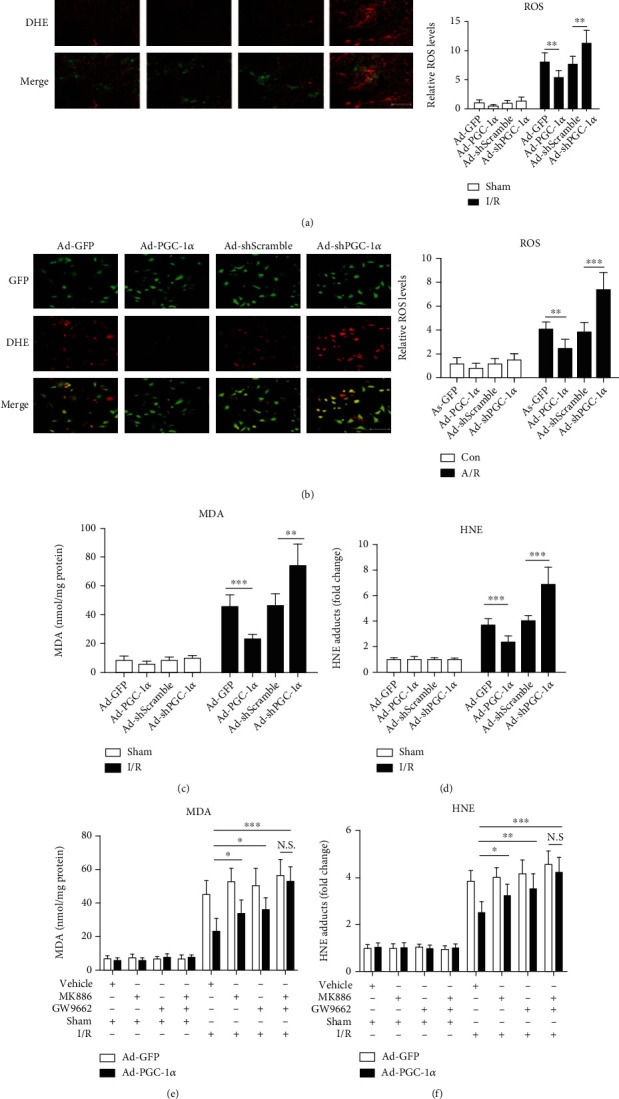
PGC-1*α* regulates the metabolism of ROS in hepatocytes in vivo and in vitro and decreases liver I/R-induced damage of oxidative stress. (a) The representative images of DHE-stained liver cryosections from mice subjected to Ad-GFP, Ad-PGC-1*α*, Ad-shScramble, and Ad-shPGC-1*α* at 1 h after liver I/R and the relative ROS levels. Bar: 200 *μ*m (*n* = 6). (b) Representative images of hepatocytes stained by DHE, and the relative ROS levels. Bar: 200 *μ*m (*n* = 3). (c) The hepatic content of MDA at 6 h after liver I/R (*n* = 6). (d) The hepatic content of HNE at 6 h after liver I/R (*n* = 6). (e) The hepatic content of MDA from mice subjected to Ad-PGC-1*α*, Ad-PGC-1*α*+MK886, Ad-PGC-1*α*+GW9662, and Ad-PGC-1*α*+MK886+GW9662 at 6 h after liver I/R (*n* = 6). (f) The hepatic content of HNE in the groups. ^∗^*P* < 0.05, ^∗∗^*P* < 0.01, and ^∗∗∗^*P* < 0.001.

It was reported that ROS was an important mediator in hepatic I/R injury [31–34]. Recently, PGC-1*α* was reported to be able to accelerate the cleaning of endogenous ROS by inducing many ROS-detoxifying enzymes [12]. Therefore, in this study, we examined the effect of PGC-1*α* on the metabolism of ROS during hepatic I/R in vivo and in vitro. ROS production was assessed by DHE staining, which is specific for superoxide anion. Our results demonstrated that PGC-1*α* was an important regulator of ROS metabolism during hepatic I/R and hepatocyte A/R injuries. As shown in Figure 6(a), ROS accumulation, as indicated by the DHE fluorescence intensities, in the livers of the Ad-GFP-transduced hepatic I/R mice was much greater than that in the related sham group, and overexpression of PGC-1*α* significantly decreased this ROS accumulation caused by hepatic I/R, whereas PGC-1*α* knockdown had a significant enhancing effect, in comparison with the Ad-GFP- or Ad-shScramble-transduced hepatic I/R mice (Figure 6(a)). Most importantly, our in vivo results were recapitulated in vitro, showing that A/R could significantly induce ROS production in the Ad-GFP-transduced hepatocytes, compared with that in the control group (Figure 6(b)). Overexpression of PGC-1*α* impaired the A/R-induced ROS production in hepatocytes, whereas PGC-1*α* knockdown had an enhancing effect (Figure 6(b)).

**Figure 7:**
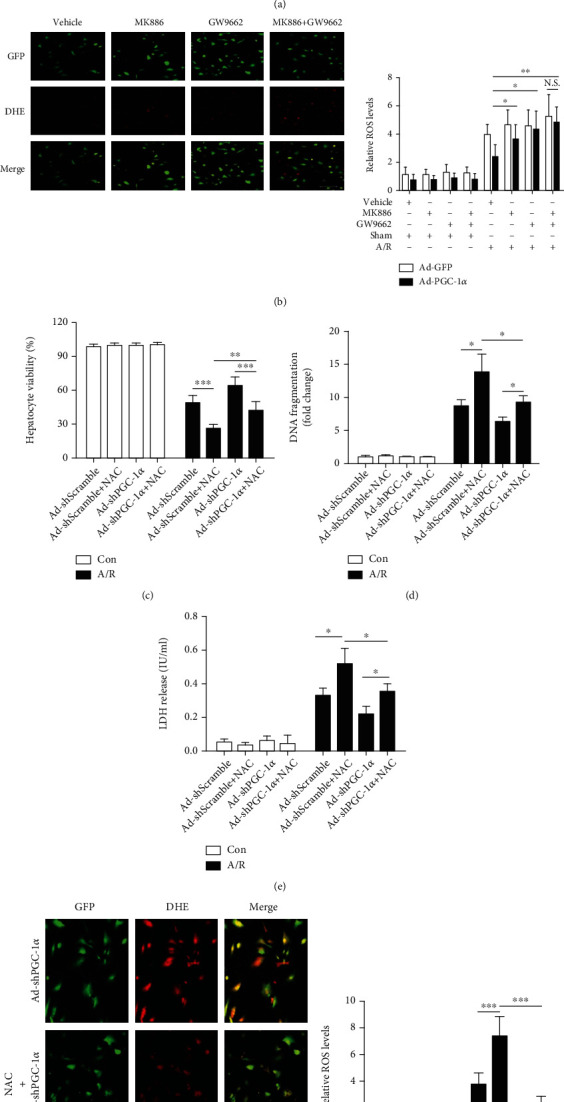
Inhibition of either PPAR*α* or PPAR*γ* alleviates the effect of PGC-1*α* on ROS production induced by liver I/R injury, and NAC pretreatment significantly reduces the aggravating effects of PGC-1*α* knockdown on A/R-induced hepatocyte injury. (a) The representative images of DHE-stained liver cryosections from mice subjected to Ad-PGC-1*α*, Ad-PGC-1*α*+MK886, Ad-PGC-1*α*+GW9662, and Ad-PGC-1*α*+MK886+GW9662, relative to the Ad-GFP control at 1 h after I/R, and the relative ROS levels. Bar: 200 *μ*m (*n* = 6). (b) The representative photographs of the DHE-stained hepatocytes subjected to Ad-PGC-1*α*, Ad-PGC-1*α*+MK886, Ad-PGC-1*α*+GW9662, and Ad-PGC-1*α*+MK886+GW9662, relative to the Ad-GFP control at 1 h after A/R, and the relative ROS levels in the hepatocytes of each group; bar: 200 *μ*m (*n* = 3). (c) The Ad-shScramble- and Ad-shPGC-1*α*-transduced hepatocytes were pretreated with NAC for 1 h before the onset of A/R, respectively. At 24 h after A/R, the cell viability was determined by a CCK-8 assay (*n* = 3). (d) DNA fragmentation was determined at 24 h after A/R by the Cell Death Detection ELISA assay (*n* = 3). (e) LDH release was measured in each group (*n* = 3). (f) The DHE staining of the Ad-shScramble- and Ad-shPGC-1*α*-transduced hepatocytes at 1 h after A/R, with or without NAC pretreatment, and the relative ROS levels. Bar: 200 *μ*m (*n* = 3). ^∗^*P* < 0.05, ^∗∗^*P* < 0.01, and ^∗∗∗^*P* < 0.001.

MDA and HNE, two important indicators of oxidative stress, were also measured. Consistent with the regulation of ROS metabolism, I/R significantly increased the contents of MDA and HNE in the liver tissues of the Ad-GFP-transduced hepatic I/R mice, compared with the related sham group. Overexpression of PGC-1*α* abrogated the I/R-induced increase in the MDA/HNE contents in the liver tissues significantly, whereas PGC-1*α* knockdown had an enhancing effect, in comparison with the Ad-GFP- or Ad-shScramble-transduced hepatic I/R group mice (Figures 6(c) and 6(d)). In addition, we also found that GW9662 and MK886, either alone or in combination, could reverse the regulatory effect of PGC-1*α* on the metabolism of ROS during hepatic I/R (Figure 7(a)) and hepatocytes A/R injuries (Figure 7(b)), similar to the findings of the MDA and HNE contents in vivo (Figures 6(e) and 6(f)), suggesting that its regulatory effect on the ROS metabolism was closely associated with the activation of PPAR*α* and PPAR*γ*.

### 3.9. Antioxidant Pretreatment Significantly Reduces the Aggravating Effects of PGC-1*α* Knockdown on A/R-Induced Hepatocyte Injury

To confirm the role of ROS regulation in the hepatoprotective effects of PGC-1*α*, the PGC-1*α*-knockdown hepatocytes were pretreated with 10 mM NAC, a well-known antioxidant [35], and then subjected to the A/R assay. As shown in Figures 7(c)–7(e), pretreatment with NAC significantly reduced the aggravating effects of the PGC-1*α* knockdown on A/R-induced hepatocyte injury, as evidenced by the increase in cell viability and the decrease in cell death, compared to the Ad-shScramble-transduced control hepatocytes (Figures 7(c)–7(e)). The ROS-scavenging effect of NAC was also confirmed by DHE staining (Figure 7(f)). Together, these data indicate that the regulation of ROS metabolism is another important hepatoprotective mechanism of PGC-1*α* in liver I/R injury.

### 3.10. PGC-1*α* Induces the Gene Expression of ROS-Detoxifying Enzymes In Vitro and In Vivo and Increases the Activities of SOD, CAT, and GPX in the Liver

**Figure 8:**
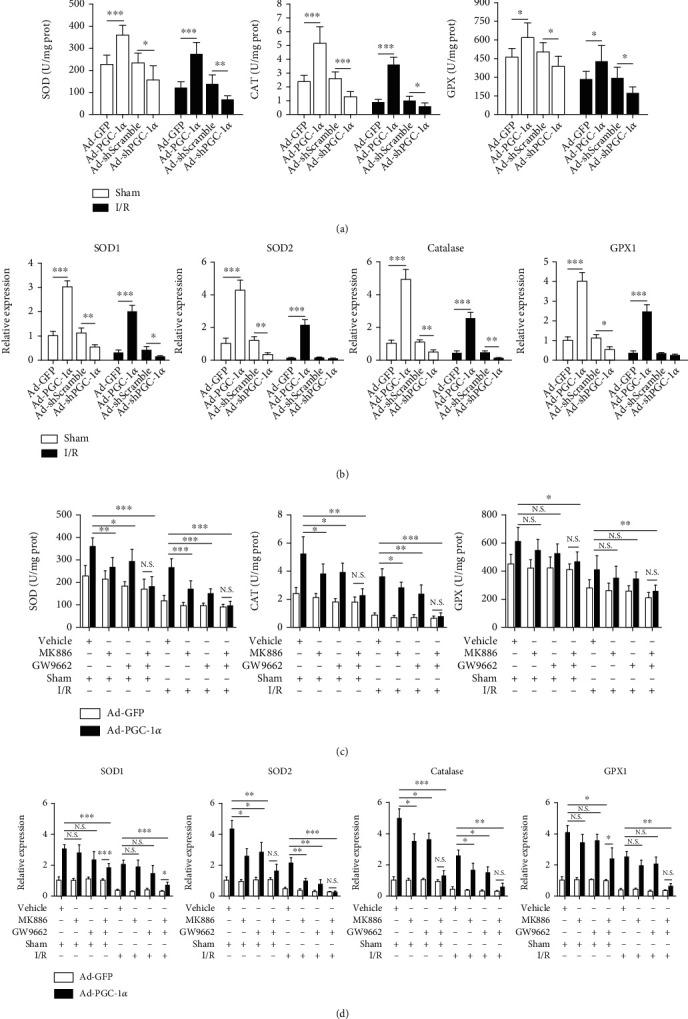
PGC-1*α* induces the gene expression of ROS-detoxifying enzymes and increase the activities of SOD, CAT, and GPX in the liver, which is associated with the activities of PPAR*α* and PPAR*γ*. (a) The activities of SOD, CAT, and GPX in the liver tissues from mice subjected to Ad-GFP, Ad-PGC-1*α*, Ad-shScramble, and Ad-shPGC-1*α* at 6 h after liver I/R (*n* = 6). (b) The relative gene expression of SOD1, SOD2, catalase, and GPX1 in the liver tissues from the mice were assessed by qRT-PCR (*n* = 6). (c) The activities of SOD, CAT, and GPX in the liver tissues from mice subjected to Ad-PGC-1*α*, Ad-PGC-1*α*+MK886, Ad-PGC-1*α*+GW9662, and Ad-PGC-1*α*+MK886+GW9662, relative to the Ad-GFP control at 6 h after liver I/R (*n* = 6). (d) The relative gene expression levels of SOD1, SOD2, catalase, and GPX1 in the liver tissues (*n* = 6). ^∗^*P* < 0.05, ^∗∗^*P* < 0.01, and ^∗∗∗^*P* < 0.001.

To further investigate the mechanisms by which PGC-1*α* reduces liver oxidative damage, we measured the activities of the main antioxidant enzymes in the liver tissues, including SOD, CAT, and GPX. As shown in Figure 8(a), among the sham groups, the contents of ROS-detoxifying enzymes were significantly upregulated by PGC-1*α* overexpression. In contrast, PGC-1*α* knockdown caused a slight decrease, in comparison with the Ad-GFP- or Ad-shScramble-transduced sham mice. As expected, I/R significantly decreased the hepatic activities of SOD, CAT, and GPX in the Ad-GFP- or Ad-shScramble-transduced mice, in comparison with the related sham group mice. Overexpression of PGC-1*α* attenuated these decreases caused by I/R, whereas PGC-1*α* knockdown had the converse effect, in comparison with the Ad-GFP- or Ad-shScramble-transduced hepatic I/R group mice (Figure 8(a)). We further investigated the molecular mechanisms involved in the PGC-1*α*-mediated increase in the activities of SOD, CAT, and GPX by quantitative RT-PCR analysis of liver tissues and primary hepatocytes, which demonstrated that hepatic I/R or hepatocytes A/R downregulated the gene expression levels of SOD1, SOD2, CAT, and GPX1 in the liver tissues (Figure 8(b)) or the hepatocytes (Supplementary Fig. S1A), respectively, in comparison with their corresponding controls. Overexpression of PGC-1*α* inhibited the downregulation of SOD1, SOD2, GPX1, and catalase expression by I/R or A/R, whereas PGC-1*α* knockdown had an enhancing effect (Figure 8(b) and Supplementary Fig. S1A). Of note, PGC-1*α* overexpression and knockdown could also cause moderate changes in the expression levels of these antioxidant enzymes in the livers of the sham group and hepatocytes under normoxia conditions (Figure 8(b) and Supplementary Fig. S1A). Intriguingly, we also found that GW9662 and MK886, either alone or in combination, could reverse the regulatory effect of PGC-1*α* on the activities of SOD, CAT, and GPX during hepatic I/R (Figure 8(c)), as well as the expression of SOD1, SOD2, CAT, and GPX1 in the liver tissues (Figure 8(d)) and the hepatocytes (Supplementary Fig. S1B). Taken together, these results indicate that PGC-1*α* induces the gene expression of ROS-detoxifying enzymes in hepatocytes and increases the hepatic activities of SOD, CAT, and GPX, which are closely associated with the activation of PPAR*α* and PPAR*γ*.

## 4. Discussion

The data presented in this study show unambiguously that PGC-1*α*, a coactivator of PPAR*α* and PPAR*γ*, is hepatoprotective during hepatic I/R injury in mice. We found that PGC-1*α* is basally expressed in normal livers and is increased by I/R, suggesting that PGC-1*α* is an I/R-related protein in the murine liver. Furthermore, overexpression of PGC-1*α* reduced hepatocyte injury induced by hepatic I/R, evidenced by the reduction in the serum levels of aminotransferases, the attenuation of histopathological alterations, and the inhibition of apoptosis, necrosis, and inflammatory response. Knockdown of endogenous PGC-1*α* increased sensitivity to I/R-triggered hepatic injury. Most importantly, these in vivo effects were further confirmed in vitro with a primary cultured mouse hepatocyte A/R assay, where PGC-1*α* overexpression rendered hepatocytes resistant to A/R-induced hepatocytic injury, whereas PGC-1*α* knockdown hepatocytes were more sensitive. Thus, at the cellular level, both loss-of-function and gain-of-function experiments reinforce the conclusion that PGC-1*α* is a positive regulator of hepatoprotection against I/R injury.

Recently, PPAR*α* and PPAR*γ* have been verified to be protective in hepatic I/R injury [3, 4, 8, 36]. PPAR*α* has been found at a high density in the liver and identified as the master regulator of hepatic lipid metabolism [12]. Moreover, PPAR*α* has been proven to be a key regulator in the inflammatory response of hepatic I/R, and C57BL/6 mice treated with the PPAR*α* agonist WY-14643 showed significantly less hepatic I/R-induced injury than mice receiving vehicle [8]. On the other hand, PPAR*γ* has been shown to be constitutively activated in hepatocytes; hepatic I/R induced significant upregulation of PPAR*γ*, and its activation with rosiglitazone or pioglitazone attenuated liver injury subjected to I/R. Conversely, mice with a greatly diminished PPAR*γ* expression/function showed more severe liver injury [3, 4]. These findings suggest that PPAR*α* and PPAR*γ* are two important endogenous regulators of ischemic liver injury. In the current study, we found that the DNA-binding activity of PPARs was reduced by liver I/R injury, and this is consistent with a previous study that reported that ischemia results in a rapid decrease in the DNA binding of PPAR*γ* [4]. Given that the activity of PPARs is widely regulated by multiple molecules, it is still reasonable that the activity of PPARs is decreased accompanied by the endogenous increase of PGC-1*α*. Furthermore, ectopic PGC-1*α* expression could indeed increase the DNA-binding activities of PPARs in hepatocytes both in vitro and in vivo, whereas knockdown of endogenous PGC-1*α* had the reverse effect. The pretreatment with a specific antagonist of either PPAR*α* (MK886) or PPAR*γ* (GW9662) could significantly abrogate he hepatoprotective effects of PGC-1a in vitro and in vivo, as evidenced by the increased levels of serum aminotransferases, inflammatory cytokines, cell death, and histological examination scores. Combination treatment of MK886 and GW9662 showed a much more significant reversal effect. These data suggested that the activation of PPAR*α* and PPAR*γ*, at least in part, was involved in the hepatoprotective effects of PGC-1*α* in hepatic I/R injury.

Previous studies have revealed that PGC-1*α* is a highly potent regulator of ROS metabolism in several cell types, whereas the excessive generation of ROS has been proven to be the main culprit in the pathogenesis of I/R injury [12, 15, 37, 38]. Therefore, in this study, we validated the regulatory role of PGC-1*α* in ROS metabolism in hepatic I/R. Our data showed that PGC-1*α* overexpression could reduce the hepatocyte ROS accumulation caused by I/R (in vivo) or A/R (in vitro) significantly, whereas the knockdown of endogenous PGC-1*α* caused a further increase in intracellular ROS levels. Accordingly, the contents of MDA and HNE, two indexes of oxidative stress, were decreased by ectopic PGC-1*α* expression during hepatic I/R in vivo, whereas the opposite effect was obtained when PGC-1*α* was downregulated. Furthermore, we found that pretreatment with NAC, a ROS scavenger, could significantly prevent the A/R-induced increased levels of ROS in Ad-shPGC-1*α*-transduced hepatocytes. Consequently, the aggravating effects of PGC-1*α* knockdown on A/R-induced hepatocyte injury was also reduced as expected, compared with the Ad-shScramble-transduced control cells. Taken together, the above data revealed that PGC-1*α* was also a powerful regulator of ROS metabolism in hepatocytes in vitro and in vivo and could effectively reduce the oxidative stress injury caused by hepatic I/R.

To explore the mechanism underlying the regulatory role of PGC-1*α* on ROS metabolism in hepatocytes, we first examined the activities of SOD, CAT, and GPX in liver tissues, which are the main antioxidant enzymes in the liver tissues. As expected, we observed that ectopic PGC-1*α* expression increased the activities of these ROS-detoxifying enzymes, whereas knockdown of endogenous PGC-1*α* reduced these activities. On the other hand, we found that PGC-1*α* regulated several important ROS-detoxifying enzymes in hepatocytes in vivo and in vitro, including SOD1, SOD2, catalase, and GPX1. This may be the molecular mechanism involved in the PGC-1*α*-induced regulation of ROS production. These findings were consistent with previous reports demonstrating that PGC-1*α* plays a key role in regulating multiple genes of the ROS defense system and is indispensable for the ROS scavenging upon oxidative stress [12, 39]. Intriguingly, we found that the pretreatment with MK886 or GW9662 significantly increased ROS production and the damage caused by oxidative stress in vivo and in vitro, which indicated that PPAR*α* and PPAR*γ* were involved in the PGC-1*α*-induced regulation of ROS production and that the regulation of ROS metabolism might be another key hepatoprotective mechanism of PGC-1*α* in liver I/R injury.

## 5. Conclusion

Given that both hepatocytes and nonparenchymal cells in the liver play essential roles in I/R injury and that Kupffer cells (KCs) also play an important role in the production of ROS and inflammatory mediators, we used gadolinium chloride (GdCl3) to eliminate KCs in mice before the liver I/R in our preliminary experiments. However, GdCl3 pretreatment did not abrogate the hepatoprotective effects of PGC-1*α* in vivo (see online Supplementary Fig. S2, 3), which indicates that the hepatocytes may be the main functional cells responsible for the role of PGC-1*α* in liver I/R injury. The role of PGC-1*α* in other nonparenchymal cells warrants further investigation. In summary, the present study demonstrates that PGC-1*α*, a member of a family of transcription coactivators, is hepatoprotective during hepatic I/R injury in mice. The mechanisms underlying its protective effects may be related to its activation of PPAR *α* and *γ*. These findings extend our understanding of the mechanisms of hepatic I/R injury, detail the important role of PGC-1*α* in this response, and suggest that PGC-1*α* is an important endogenous regulator of, and potential therapeutic target for, ischemic liver injury.

## Data Availability

No data were used.

## Abbreviations

PPARs: Peroxisome proliferator-activated receptors
PGC-1*α*: PPAR*γ* coactivator-1*α*
ROS: Reactive oxygen species
I/R: Ischemia/reperfusion
IP: Intraperitoneal injection
A/R: Anoxia/reoxygenation
ALT: Alanine aminotransferase
AST: Aspartate aminotransferase
ELISA: Enzyme-linked immunosorbent assay
TUNEL: Terminal deoxynucleotidyl transferase-mediated dUTP nick-end labeling
MDA: Malondialdehyde
HNE: 4-Hydroxynonenal
SOD: Superoxide dismutase
CAT: Catalase
GPX: Glutathione peroxidase
NAC: N-acetyl-L-cysteine
LDH: Lactate dehydrogenase.

## Supplementary Materials

Supplementary Materials Supplementary Figure 1: PGC-1*α* induces the gene expression of ROS-detoxifying enzymes in hepatocytes, which is associated with the activities of PPAR*α* and PPAR*γ*. (A) The relative gene expression levels of SOD1, SOD2, catalase, and GPX1 in hepatocytes subjected to Ad-PGC-1*α*, Ad-shPGC-1*α*, and the related control were detected by quantitative RT-PCR. (B) The relative gene expression levels of SOD1, SOD2, catalase, and GPX1 in Ad-PGC-1*α*, Ad-PGC-1*α*+MK886, Ad-PGC-1*α*+GW9662, Ad-PGC-1*α*+MK886+GW9662, relative to the Ad-GFP control at 24 h after A/R. ^∗^*P* < 0.05, ^∗∗^*P* < 0.01, and ^∗∗∗^*P* < 0.001. Supplementary Figure 2: gadolinium chloride (GdCl3) was used to eliminate KCs in mice before liver I/R. Representative photographs (200x magnification) of F4/80-stained liver sections in GdCl3 and saline pretreated mice. Supplementary Figure 3: GdCl3 pretreatment did not abrogate the hepatoprotective effects of PGC-1*α* in vivo. (A) Serum levels of ALT were assessed between GdCl3 and saline pretreated mice which had subjected to Ad-GFP, Ad-PGC-1*α*, Ad-shScramble, and Ad-shPGC-1*α*, at 6 h after liver I/R. (B) Serum levels of AST were assessed in the mice. ^∗∗∗^*P* < 0.001. Supplementary Table 1: the TaqMan primer/probe sets utilized in this study were from Applied Biosystems.
