# Supplementary material for: PGC-1α Protects against Hepatic Ischemia Reperfusion Injury by Activating PPARα and PPARγ and Regulating ROS Production
Source: Oxid Med Cell Longev. 2021 May 19;2021:6677955. doi: 10.1155/2021/6677955 (PMC8159639; doi:10.1155/2021/6677955)
Supplement: Supplementary Materials — Supplementary Figure 1: PGC-1α induces the gene expression of ROS-detoxifying enzymes in hepatocytes, which is associated with the activities of PPARα and PPARγ. (A) The relative gene expression levels of SOD1, SOD2, catalase, and GPX1 in hepatocytes subjected to Ad-PGC-1α, Ad-shPGC-1α, and the related control were detected by quantitative RT-PCR. (B) The relative gene expression levels of SOD1, SOD2, catalase, and GPX1 in Ad-PGC-1α, Ad-PGC-1α+MK886, Ad-PGC-1α+GW9662, Ad-PGC-1α+MK886+GW9662, relative to the Ad-GFP control at 24 h after A/R. ∗P < 0.05, ∗∗P < 0.01, and ∗∗∗P < 0.001. Supplementary Figure 2: gadolinium chloride (GdCl3) was used to eliminate KCs in mice before liver I/R. Representative photographs (200x magnification) of F4/80-stained liver sections in GdCl3 and saline pretreated mice. Supplementary Figure 3: GdCl3 pretreatment did not abrogate the hepatoprotective effects of PGC-1α in vivo. (A) Serum levels of ALT were assessed between GdCl3 and saline pretreated mice which had subjected to Ad-GFP, Ad-PGC-1α, Ad-shScramble, and Ad-shPGC-1α, at 6 h after liver I/R. (B) Serum levels of AST were assessed in the mice. ∗∗∗P < 0.001. Supplementary Table 1: the TaqMan primer/probe sets utilized in this study were from Applied Biosystems. [file 6677955.f1.doc]

**Supplementary Materials and Methods.**

**Animals.**

Male 8-week-old C57BL/6 (18–20 g) mice were supplied by the Animal Research Center at the First Clinical Medical School of Harbin Medical University (Harbin, China). All surgical procedures and care administered to the animals were approved by the institutional ethic committee, and this study also complied with the criteria in Guide for the Care and Use of Laboratory Animals.

**Construction of adenoviral (Ad) vectors.**

The adenoviral vector for the expression of PGC-1α (Ad-PGC-1α) or GFP alone (Ad-GFP) was a kind gift from Daniel P. Kelly (Washington University School of Medicine, St. Louis, MO). We generated the PGC-1α shRNA adenoviral vector (Ad-shPGC-1α) using an effective sequence (5′ GGTGGATTGAAGTGGTGTAGA 3′) and also its negative control Ad-shScramble with sequence (5′ AACAGTCGCGTT TGCGACTGG 3′) that does not match any known mammalian GENEBANK sequence, as described before (13-14). The recombinant adenoviruses were propagated in HEK293 cells, and purified by cesium chloride density gradient centrifugation. The infectious viral titer was determined by measuring the median tissue culture infective dose (TCID50).

**Adenoviral vectors transduction.**

To achieve the goals of intrahepatic PGC-1α overexpression and knockdown, Ad-PGC-1α and Ad-shPGC-1α were delivered to mice by tail-vein injection with 1×10^9^ TCID50/mouse, respectively, as described previously [30-32]. Ad-GFP and Ad-shScramble were used as a negative control, respectively. After 72h, the liver I/R procedures were carried out. For the in vitro experiments, primary hepatocytes were cultured with Ad-PGC-1α, Ad-shPGC-1α, Ad-GFP or Ad-shScramble for 2 h at MOI of 80, and the functional assays were carried out 48h later. To verify the efficiency of transduction, parts of the collected liver tissues were fixed in 4% paraformaldehyde followed by an overnight incubation in 30% sucrose incubation and then embedded in OCT compound for GFP visualization. Fluorescence microscopy was used to document GFP expression in these adenoviral vectors treated hepatocytes and liver tissues.

**Gadolinium chloride treatment**

Mice received either gadolinium chloride (Sigma-Aldrich) dissolved in normal saline (10 mg/kg) or normal saline alone by i.v. injection 24 h before ischemia. Kupffer cell depletion was confirmed by immunostaining of liver sections using the anti-F4/80 antibody.

**Mouse hepatic I/R model.**

We used a previously characterized mouse model of partial hepatic I/R injury [20]. In brief, the mice were anesthetized by intraperitoneal (IP) injection of sodium pentobarbital (60 mg/kg), and a midline laparotomy was performed. Then, the left lateral and median lobes of the liver were clamped at its base using an atraumatic clip. Throughout anesthesia, body temperature was monitored by a rectal probe and maintained at 37°C by a heating lamp. After 75 min of ischemia, the clip was removed, initiating hepatic reperfusion. Sham-operated mice underwent the same procedure, but without vascular occlusion. In certain groups, animals were infused 1.5h prior to the onset of liver ischemia with a single dose of MK886 (a special PPARα antagonist (3mg/kg, IP) or GW9662 (a special PPARγ antagonist, 1mg/kg, IP), both of these antagonists were dissolved in DMSO/saline. I/R control groups received saline solution only with DMSO. MK886, GW9662 and DMSO were obtained from Sigma Chemical (St. Louis, MO, USA). Mice were sacrificed at 6h after reperfusion, liver and serum samples were collected for analysis.

**Measurement of parameters in sera.**

The levels of ALT and AST in sera were measured with an autobiochemical analyzer (Toshiba, TBA-200FR), as described previously [33]. The serum levels of TNF-α (TNF-α), IL-1β (interleukin-1β), IL-6 (interleukin-6) and MIP-2 (macrophage inflammatory protein 2) were measured with enzyme-linked immunosorbent assay (ELISA) kits (all R&D Systems, Minneapolis, MN), according to the manufacturer’s instructions.

**Histological examination.**

Liver specimens were fixed in 10% buffered formalin, embedded in paraffin, stained with hematoxylin and eosin (H&E), and examined with a light microscope. The histopathological scoring analysis was performed blindly according to previously described methods [33]. The assessment was expressed as the sum of the individual score grades from 0 (no findings), 1 (mild), 2 (moderate), to 3 (severe) for each of the following six parameters: cytoplasmic color fading, vacuolization, nuclear condensation, nuclear fragmentation, nuclear fading, and erythrocyte stasis.

**Electrophoretic mobility shift assay (EMSA).**

Nuclear extracts (NE) of liver tissue and primary hepatocytes were prepared as described previously [34]. DNA-binding activity was confirmed with biotin-labeled oligonucleotide bio-PPARs probe (5’- CAAAACTAGGTCAAAGGTCA-3’) using an EMSA kit according to the manufacturer’s instructions (Viagene, Beijing, China). The probe was resolved on a 4% polyacrylamide gel containing 0.25 × TBE (Tris/borate/EDTA) buffer, and visualized with a CoolImger imaging system (IMGR002, Viagene, Beijing, China). Where indicated, specific competitor (SC) or nonspecific competitor (NSC) oligonucleotide was added before the labeled probe and incubated for 10 min on ice. For supershift analysis, 1μg of either PPARα or PPARγ antibody (Santa Cruz Biotechnology) was added following an initial 20 minutes of incubation with the biotin-labeled probe. The incubation was then continued for another 20 minutes prior to electrophoresis. Sonicated salmon sperm DNA was used as nonspecific competitor.

**Western blotting analysis.**

Protein lysates of liver or primary hepatocytes were prepared, separated onto SDS-polyacrylamide gels and transferred to PVDF membrane as previously described [32]. Western blotting was performed using appropriate primary antibodies and horseradish peroxidase-conjugated suitable secondary antibodies, followed by detection with enhanced chemiluminescence (Pierce Chemical). GAPDH was used as protein loading control, and the levels of proteins were normalized with respect to GAPDH band density. The antibody against cleaved PARP was purchased from Cell Signaling Technology (Cat. #9542, Danvers, MA, USA). The anti-GAPDH was purchased from Kangchen Bio-tech (Cat. KC-5G4, Shanghai, China). Anti-PGC-1α Mouse mAb was purchased from Calbiochem (Cat. No. ST1202, San Diego, CA, USA).

**Terminal deoxynucleotidyl transferase-mediated dUTP nick-end labeling (TUNEL) assay.**

DNA fragments in liver sections, resulting from oncotic necrosis and apoptosis were detected by TUNEL method (In situ cell death detection kit, Roche Applied Science), as described previously [33]. TUNEL positive cells were counted in 10 HPF/section under light microscopy (x400) and expressed as a percentage of the total hepatocytes.

**DNA fragmentation ELISA**

The quantitative determinations of cytoplasmic histone-associated-DNA-fragmentation (mono and oligonucleosomes) due to cell death were measured using the Cell Death Detection ELISA kit (Roche Diagnostics GmbH, Indianapolis, IN), according to the manufacturer’s protocol. The experiments were repeated in triplicates.

**Caspase-3 activity assay**

Caspase-3 activity was performed using Caspase-3 Cellular Activity Assay Kit (Calbiochem). Liver tissue sample and cell lysis were used according to the manufacturer’s instruction.

**Hepatocytes isolation.**

Mouse hepatocytes were isolated by a modified in situ collagenase perfusion technique as described previously [20]. Viability and purity of cells were determined by trypan blue exclusion.

**A/R and cell death assay.**

To simulate tissue I/R, hepatocytes were treated as described previously [20]. Briefly, after overexpression or knockdown of PGC-1α, the hepatocytes were incubated at 37°C in Krebs-Ringer-hydroxyethylpiperazine-N-2 ethanesulfonic acid (KRH) buffer at pH 6.2 in an anaerobic chamber for 4 hours. To simulate reoxygenation and return to physiological pH during reperfusion, anaerobic KRH (pH 6.2) was replaced with aerobic KRH (pH 7.4) at the beginning of reoxygenation. In certain groups, the hepatocytes also pretreated with MK886 (10μM) or GW9662(5μM), 1 h before the onset of A/R. 24 h after A/R, live cells were first examined by fluorescence microscopy, then the cell viability was measured with Cell Counting Kit-8 (CCK-8, Dojindo Molecular Technologies, CK04–13) according to the instructions of the manufacturer. Cell cytotoxicity or necrosis was assessed by measuring lactate dehydrogenase (LDH) release into the medium by necrotic cells using an LDH Cytotoxicity Assay Kit (Promega, Madison, WI, USA). The experiments were repeated in triplicates.

**ROS detection.**

The cellular and tissues ROS levels were estimated as described previously [33]. ROS, particularly superoxide, oxidizes the non-fluorescent probe dihydroethidium (DHE) to ethidium bromide, which is excited at 488 nm with an emission of 610 nm. The intensity of ethidium bromide fluorescence is proportional to the capacity of superoxide generation in the tissue. 30 min after reperfusion, fresh liver cryosections (10 μm) were prepared from 3 separate livers of every group, and stained with 5 μM DHE in PBS at 37°C in a light protected, humid chamber for 30 min, then rinsed extensively with PBS, coverslipped and digitally imaged with a fluorescent microscope. For detecting the cellular levels of ROS in hepatocytes A/R assay, the cells were incubated with 5 μM DHE in KRH buffer (pH 7.4) at 37°C in the dark for 30 min and washed with KRH buffer before fluorescent detection. The relative ROS levels, which are proportional to the fluorescence intensity, were quantified using Image-Pro Plus software. DHE was purchased from Molecular Probes (Eugene, OR, USA)

**Hepatic 4-hydroxynonenal (4-HNE) content**

Lipid peroxides are unstable indicators of oxidative stress in cells that decompose to form more complex and reactive compounds such as 4-HNE, which has been shown to be capable of binding to proteins and forming stable HNE adducts. Hepatic HNE content was determined using a kit (Cell Biolabs, San Diego, CA). In brief, BSA or hepatic tissue extracts (10μg/mL) are adsorbed onto a 96-well plate for 12 hrs at 4°C. HNE adducts present in the sample or standard are probed with anti-HNE antibody, followed by an HRP conjugated secondary antibody. The HNE-protein adducts content in an unknown sample is determined by comparing with a standard curve.

**MDA determination.**

The levels of MDA in liver tissues were determined by the thiobarbituric acid method using an assay kit from Nanjing Jiancheng Bioengineering Institute (Nanjing, China), as described previously [33], and the liver homogenate protein was measured by the CBB method. The content of MDA was expressed as nmol per mg protein and calculated as follows:

MDA concentration (nmol/mgprot) = [(measuring tube OD−control tube OD)/(standard tube OD−blank tube OD)]×standard substance concentration÷protein concentration

**Measurement of the activities of SOD, CAT and GPX.**

The activities of SOD, CAT and GPX were determined with commercially available assay kits from Nanjing Jiancheng Bioengineering Institute (Nanjing, China), as described previously [33, 37]. Briefly, homogenate protein concentration in the liver was measured by the CBB method. The T-SOD activity was measured as that which will inhibit the rate of oxidation of hydroxylamine by 50% in a coupled system, using xanthine and xanthine oxidase at 37 °C in 1.0 mg/mL protein concentration of liver tissue homogenate. The CAT activity was measured by the rate of disappearance of H_2_O_2_ at 240 nm and expressed as micromoles of H_2_O_2_ decomposed per minute per gram of liver tissue. The GPX activity is the amount of enzyme that will oxidize 1 μmol/L GSH in the reaction system at 37 °C per minute in 1 mg of liver tissue. The activities of these parameters were expressed as units of nitrite per mg protein. All samples were measured in triplicate.

**Statistics Analysis.**

All data are expressed as mean ±SD. Significant differences between groups were determined by ANOVA, with a Bonferroni correction for continuous variable and multiple groups. Two-tailed Student’s t test was used for the comparison of a normally distributed continuous variable between 2 groups. The level of significance was set at a P value less than 0.05 for all analyses.

**Supplementary Figure S1**


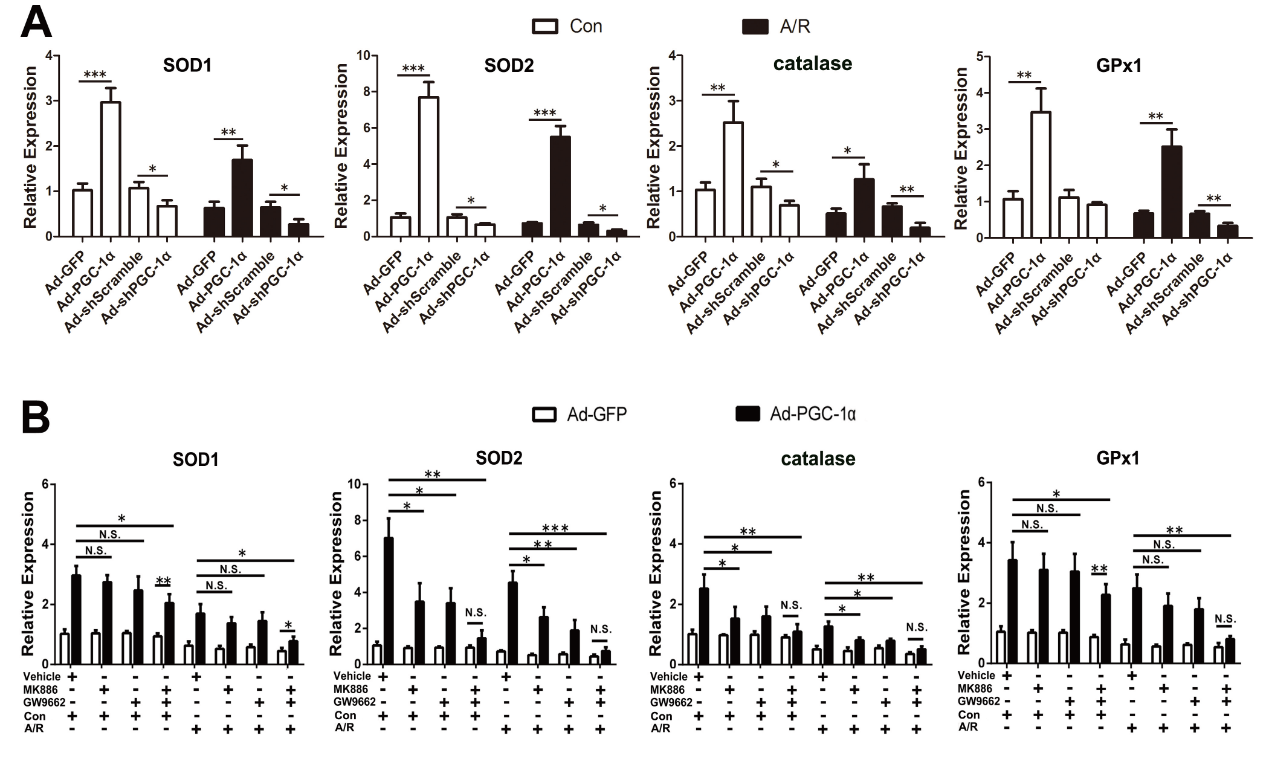


**Supplementary Figure 1.** PGC-1α induces the gene expression of ROS-detoxifying enzymes in hepatocytes, which is associated with the activities of PPARα and PPARγ. (A) The relative gene expression levels of SOD1, SOD2, catalase and GPx1 in hepatocytes subjected to Ad-PGC-1α, Ad-shPGC-1α and the related control were detected by quantitative RT-PCR; (B) The relative gene expression levels of SOD1, SOD2, catalase and GPx1 in Ad-PGC-1α, Ad-PGC-1α + MK886, Ad-PGC-1α + GW9662, Ad-PGC-1α + MK886 + GW9662, relative to the Ad-GFP control at 24 h after A/R. *P < 0.05, ** P < 0.01, *** P < 0.001.

**Supplementary Figure S2**


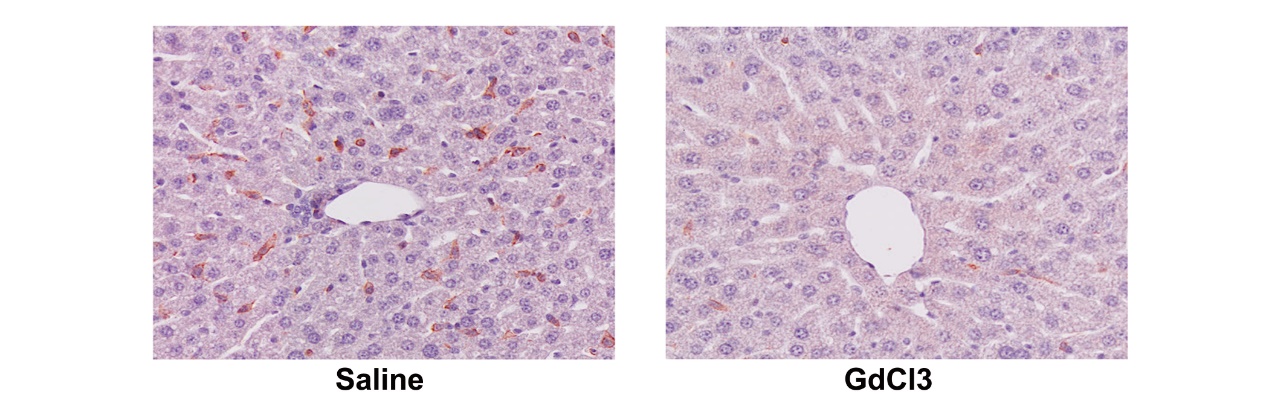


**Supplementary Figure 2.** Gadolinium chloride (GdCl3) was used to eliminate KCs in mice before liver I/R. Representative photographs (200×magnification) of F4/80-stained liver sections in GdCl3 and saline pretreated mice.

**Supplementary Figure S3**


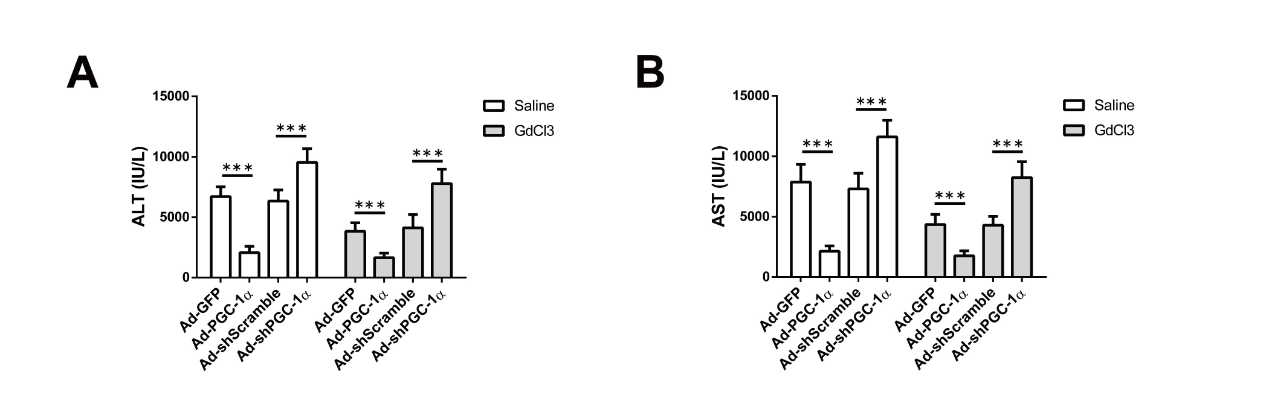
**Supplementary Figure 3.** GdCl3 pretreatment did not abrogate the hepatoprotective effects of PGC-1α in vivo. (A) Serum levels of ALT were assessed between GdCl3 and saline pretreated mice which had subjected to Ad-GFP, Ad-PGC-1α, Ad-shScramble and Ad-shPGC-1α, at 6 h after liver I/R; (B) Serum levels of AST were assessed in the mice. *** P < 0.001.

**Supplementary Table 1.**

The TaqMan Primer/Probe Sets utilized in this study were from Applied Biosystems.

| Gene | Gene ID | Assay ID |
| --- | --- | --- |
| SOD1 | [20655](http://www.ncbi.nlm.nih.gov/gene?term=20655) | Mm01344233_g1 |
| SOD2 | [20656](http://www.ncbi.nlm.nih.gov/gene?term=20656) | Mm00449726_m1 |
| GPx1 | [14775](http://www.ncbi.nlm.nih.gov/gene?term=14775) | Mm00656767_g1 |
| CAT | [12359](http://www.ncbi.nlm.nih.gov/gene?term=12359) | Mm00437992_m1 |
| TNF-α | [21926](http://www.ncbi.nlm.nih.gov/gene?term=21926) | Mm00443258_m1 |
| IL-1β | 16176 | [Mm01336189_m1](https://www.thermofisher.com/taqman-gene-expression/product/Mm01336189_m1?CID=&ICID=&subtype=) |
| IL-6 | [16193](http://www.ncbi.nlm.nih.gov/gene?term=16193) | Mm00446190_m1 |
| MIP-2 | [20310](http://www.ncbi.nlm.nih.gov/gene?term=20310) | Mm00436450_m1 |
| GAPDH | [14433](http://www.ncbi.nlm.nih.gov/gene?term=14433) | Mm99999915_g1 |
